# An under-ice bloom of mixotrophic haptophytes in low nutrient and freshwater-influenced Arctic waters

**DOI:** 10.1038/s41598-021-82413-y

**Published:** 2021-02-03

**Authors:** Dorte H. Søgaard, Brian K. Sorrell, Mikael K. Sejr, Per Andersen, Søren Rysgaard, Per Juel Hansen, Annaliina Skyttä, Signe Lemcke, Lars Chresten Lund-Hansen

**Affiliations:** 1 Greenland Climate Research Centre (C/O Greenland Institute of Natural Resources), Kivioq 2, Box 570, 3900 Nuuk, Greenland; 2 Department of Biology, Arctic Research Centre, Aarhus University, Ole Worms Allé 1, Build. 1135, 8000 Aarhus C, Denmark; 3 Department of Bioscience, Aarhus University, Vejlsøvej 25, 8000 Silkeborg, Denmark; 4 Centre for Earth Observation Science, CHR Faculty of Environment Earth and Resources, University of Manitoba, Winnipeg, MB Canada; 5 Faculty of Science, Marine Biological Section, University of Copenhagen, Strandpromenaden 5, 3000 Helsingør, Denmark; 6 Faculty of Biological and Environmental Sciences Research Infrastructure-Core Facilities, University of Helsinki, Fabianinkatu 33, P.O. Box 3, 00014 Helsinki, Finland

**Keywords:** Ecology, Microbiology, Biogeochemistry, Climate sciences, Ecology

## Abstract

The pelagic spring bloom is essential for Arctic marine food webs, and a crucial driver of carbon transport to the ocean depths. A critical challenge is understanding its timing and magnitude, to predict its changes in coming decades. Spring bloom onset is typically light-limited, beginning when irradiance increases or during ice breakup. Here we report an acute 9-day under-ice algal bloom in nutrient-poor, freshwater-influenced water under 1-m thick sea ice. It was dominated by mixotrophic brackish water haptophytes (*Chrysochromulina*/ *Prymnesium*) that produced 5.7 g C m^−2^ new production. This estimate represents about half the annual pelagic production, occurring below sea ice with a large contribution from the mixotrophic algae bloom. The freshwater-influenced, nutrient-dilute and low light environment combined with mixotrophic community dominance implies that phagotrophy played a critical role in the under-ice bloom. We argue that such blooms dominated by potentially toxic mixotrophic algae might become more common and widespread in the future Arctic Ocean.

## Introduction

Satellite-based remote sensing data suggest that annual pelagic net primary production in shelf areas of the Arctic Ocean has increased by 20% from 1998 to 2009, mainly due to the longer seasonal duration of the ice-free period, and thus a longer pelagic growth season^1^. The contribution of phytoplankton productivity beneath ice-covered oceans to annual pelagic net primary production was until recently thought to be negligible, primarily because of the strong light attenuation by snow and sea ice^2^. However, this view has recently been challenged by observations of extensive diatom-dominated under-ice blooms beneath thick melting sea ice or under refrozen leads^3–5^. In these studies, diatom- and *Phaeocystis*-dominated under-ice blooms were triggered by increased under-ice irradiance due to melting sea ice, refrozen leads and/or melt pond formation (i.e. increased transmittance) and were fuelled by an excess of nutrients in the under-ice waters^3–7^. However, an improved understanding of the role, extent and frequency of these extensive under-ice blooms is needed to describe and model future changes in annual pelagic net primary productivity in the Arctic Ocean.

The Young Sound fjord in NE Greenland is covered by sea ice for most of the year (8–10 months)^8^ and has a pronounced summer stratification that impedes nutrient supply from deeper waters^9,10^. The combination of light limitation by sea ice and terrestrial run-off and low nutrient supply in Young Sound is responsible for its low annual pelagic net primary productivity (10.3 g C m^−2^ year^−1^)^8,11^, which is low compared to the more productive Godthåbsfjord on the southwest coast of Greenland (between 84.6 and 139.1 g C m^−2^ year^−1^)^12^. The future annual pelagic net primary production in Young Sound and across the Arctic Ocean is likely to increase overall as the ice-free season lengthens. Counteracting the effects of a longer pelagic growing season is, however, the recent increase in freshwater fluxes from Arctic glaciers and the general freshening of the Young Sound fjord, adjacent fjords^13,14^ and the Arctic Ocean^15,16^. This freshening may lead to an intensified stratification and therefore a weakened vertical supply of nutrients^9,17^.

In this study, we demonstrate for the first time an under-ice bloom driven by mixotrophic brackish-water haptophytes beneath 1-m thick sea ice in a nutritionally dilute environment. These haptophyte genera (*Prymnesium* and *Chrysochromulina*) can be highly toxic to fish and cause severe damage to the aquaculture industry, e.g. in Norway^18,19^. Harmful blooms of *Chrysochromulina* spp. have been observed in the Skagerrak/Kattegat^18–20^, the Baltic Sea^21^, and in Resolute Bay, Canada^22^, but this is, to our knowledge, the first observation of a high Arctic under-ice bloom driven by mixotrophic haptophytes.

Species of *Prymnesium* and *Chrysochromulina* are mixotrophs, combining photosynthesis and prey uptake. This provides them with a competitive advantage in light- and nutrient-limited environments^23–28^. Predation/grazing provides mixotrophic *Chrysochromulina* species with organic carbon as an energy source as well as additional nitrogen, allowing them to grow when light is limiting photosynthesis or dissolved inorganic nutrients in the sea ice and seawater are low^25–28^. The concentration of haptophytes in the present bloom (~ 20 × 10^5^ cells l^−1^) was similar to those cell concentrations found during the peak of harmful *Chrysochromulina* blooms in the Skagerrak in southern Norway^18,19^. The indication of a mixotrophic-based bloom suggests that mixotrophic algae may play an important role in driving the Arctic spring bloom and thus the ecosystem and carbon dynamics in this area. Some haptophytes tend to be more abundant in less saline waters, as exemplified by records of *Chrysochromulina spp.* in the low saline (salinity 5–6) Baltic Sea and in the Kattegat (salinity 15–25)^19–21^. This further implies that mixotrophic-driven harmful algae blooms might become more common and widespread in a more freshwater-influenced future Arctic Ocean.

## Results and discussion

### Sea ice and water column properties

In this study, we document that a mixotrophic-driven under-ice bloom can be an important seasonal feature of Arctic fjords. An improved insight into the extent and frequency of these extensive blooms driven by potentially mixotrophic haptophyte species underneath thick sea ice is fundamental for understanding and modelling future changes in Arctic Ocean pelagic net primary productivity. If blooms of potentially toxic algae become more common in the Arctic, this might have a large ecological and socio-economic impact.

**Figure 1:**
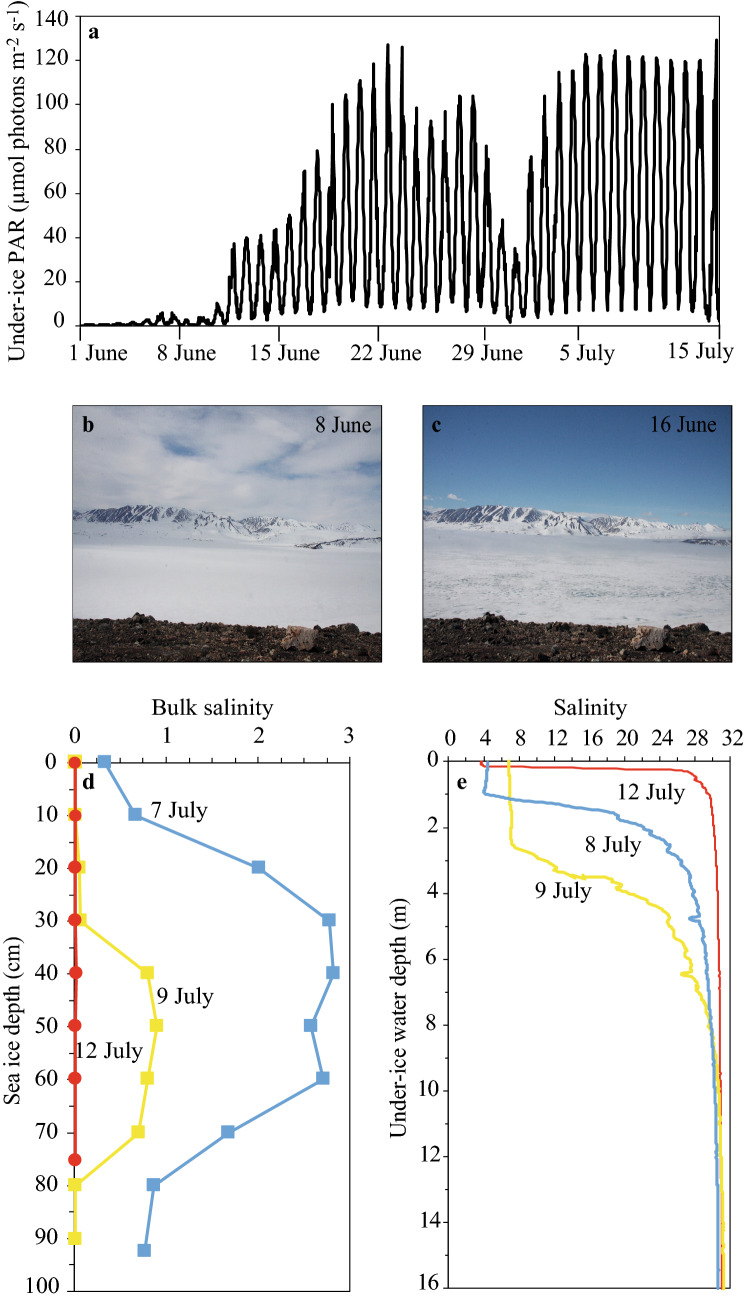
Physico-chemical properties of sea ice and seawater. Development of (**a**) under ice irradiance (PAR, µmol photons m^−2^ day^−1^), (**b**) melt pond coverage on sea ice (~ 0%), (**c**) melt pond coverage on sea ice (~ 10%), (**d**) sea ice bulk salinity and (**e**) under-ice water salinity in Young Sound, NE Greenland, during the sea ice melting season in 2017.

The bloom was initiated in nutrient-poor brackish water under 1-m thick sea ice covered with melt ponds in the Young Sound fjord in Northeast Greenland. Surface melt ponds started to form on the sea ice from snow meltwater on 11 June 2017 (Fig. 1). Consequently, this period was characterized by a continuous increase in maximum under-ice PAR from 4.0 µmol photons m^−2^ s^−1^ before melt pond formation (8 June 2017; Fig. 1b,c), to a daily maximum of 127.3 µmol photons m^−2^ s^−1^ on 23 June, and similar on 15 July at ice break-up (Fig. 1a).

The continuous increase in melt pond coverage (maximum of ~ 40%) and sea ice thaw (from 0.95 to 0.75 m thickness) resulted in a nearly full desalination of the sea ice towards 12 July and a concomitant decrease in under-ice salinity on 9 July (Fig. 1d,e). The sea ice cover broke up on 15 July after which the ice floes were transported out of the fjord by winds and the tide.

### Spring bloom initiated by mixotrophic haptophytes

**Figure 2:**
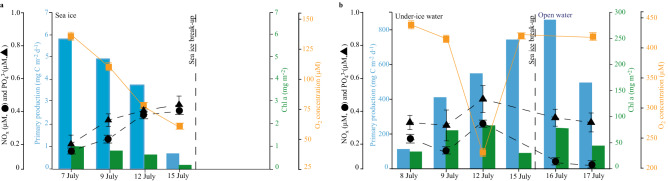
Comparison of sea ice and seawater biomass and productivity. Temporal development of (**a**) sea ice integrated primary production in mg C m^−2^ day^−1^ (blue bars), integrated Chl *a* in mg m^−2^ (green bars), NO_x_ concentration in µM (black circles), PO_4_^3-^ concentration in µM (black triangles) and O_2_ bulk concentration in µM (orange squares) and (**b**) under-ice and open water integrated primary production in mg C m^−2^ day^−1^ (blue bars), integrated Chl *a* in mg m^−2^ (green bars), NO_x_ concentration in µM at 1 m (black circles), PO_4_^3-^ concentration in µM at 1 m (black triangles) and O_2_ bulk concentration in µM at 1 m (orange squares). The data points represent the average of triplicate samples; error bars indicate SD of the mean.

**Figure 3:**
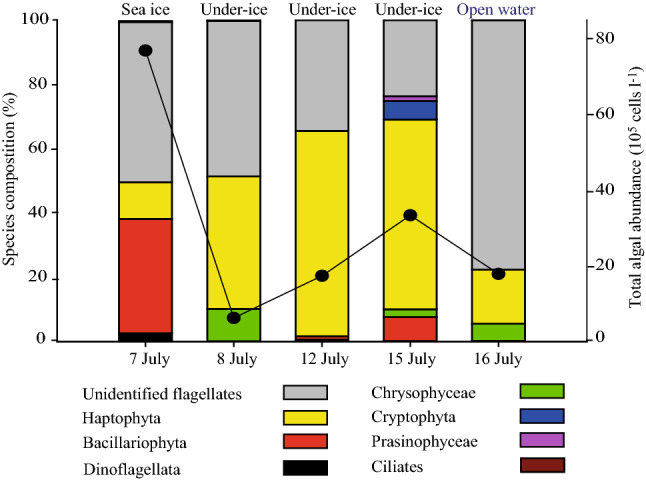
Algal community composition. Temporal development of algal species composition (%) in the entire Sea ice column and under-ice water at 1 m (bars) and the total algal abundance (black circles).

**Figure 4:**
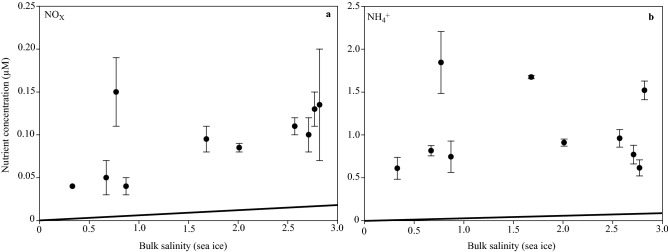
Sea ice nutrient concentrations in relation to salinity. Concentrations of (**a**) NO_x_ and (**b**) NH_4_^+^ versus bulk salinity in sea ice prior to the complete brine drainage event. The solid line indicates the expected dilution line predicted from salinity and nutrient concentrations in seawater (15 m depth, salinity of 33). Values below the dilution line indicate nutrient depletion; values above it indicates either production or net accumulation. Data points represents treatment mean ± SD (n = 3).

Prior to the complete brine drainage from melt ponds and ice melt towards 12 July, primary productivity and algal biomass in the sea ice were five to ten-fold higher, respectively (up to 5.8 mg C m^−2^ day^−1^ and 1.0 mg Chl *a* m^−2^) (blue and green bars in Fig. 2a) on 7 July than after the drainage event on 15 July (sea ice algal productivity of 0.6 mg C m^−2^ day^−1^ and algal biomass of 0.2 mg Chl *a* m^−2^; blue and green bars in Fig. 2a). Furthermore, the total algal abundance in the sea ice on 7 July was 12-fold higher (up to 77 × 10^5^ cells l^−1^) than the under-ice total algal abundance on 8 July (up to 6.2 × 10^5^ cells l^−1^) (black line in Fig. 3). Based on bulk nutrient concentrations and bulk salinity in the sea ice, and the expected dilution line, we have calculated whether nutrients were depleted or whether production or net accumulation of nutrients had occurred at this site (Fig. 4). Sea ice nutrient-bulk salinity plots indicate accumulation of algal nutrients (NO_x_ and NH_4_^+^) in the sea ice, which indicates that heterotrophic activity may have played an important role in nutrient re-cycling (Fig. 4). This is supported by the heavily undersaturated O_2_ concentrations in the sea ice (Fig. 2a, solid orange line). Together, these results suggest that the ice-associated heterotrophic productivity largely exceeded ice-associated primary productivity in this late season sea ice.

The complete drainage event towards 12 July marked the termination of the sea ice photosynthetic activity (sea ice algal biomass of 0.2 mg Chl *a* m^−2^; green bars in Fig. 2a). Therefore, the remaining sea ice algal community and algal nutrients present in the ice during melt likely sloughed from the sea ice to the under-ice seawater during the complete brine drainage towards 12 July. PAM fluorescence measurements verified that the drainage event ended the sea ice photosynthetic activity in the brine as demonstrated by a rapid decrease in maximum quantum yield (*F*_*v*_*/F*_*m*_) of the sea ice from 0.21 ± 0.07 on 9 July to 0.11 ± 0.04 on 12 July (Table 1).

**Table 1:** Algal productivity and photobiology.

Sampling date	Under-ice and open water		Sea ice	
	Biomass specific productivity	*F*_v_/*F*_m_	Biomass specific productivity	*F*_v_/*F*_m_
7 July	–	–	5.53	0.22 ± 0.03
8 July	4.40	0.24 ± 0.04	–	–
9 July	5.80	0.12 ± 0.02	5.87	0.21 ± 0.03
12 July	6.25	0.13 ± 0.01	5.61	0.11 ± 0.04
15 July	25.40	0.35 ± 0.10	3.95	0.13 ± 0.05
16 July	10.96	0.21 ± 0.07	–	–
17 July	10.60	0.22 ± 0.06	–	–

Biomass specific productivity (mg C mg Chl *a*^−1^ day^−1^) and algal photobiology from 7 to 17 July in under-ice water (1 m), open seawater (1 m) and sea ice.

Data points represents treatment mean ± SD (n = 3).

These numbers are averages for the sectioned (10 cm) entire ice cores, and support the hypothesis that the complete brine drainage terminated the sea ice photosynthetic activity. Nevertheless, we observed a dramatic increase in under-ice water maximum quantum yield (Fv/Fm), biomass specific productivity and total algal abundance from 0.12 ± 0.02, 5.80 mg C mg Chl *a*^−1^ day^−1^ and 6.2 × 10^5^ cells l^−1^ on 9 July to a maximum of 0.35 ± 0.10, 25.40 mg C mg Chl *a*^−1^ day^−1^ and 34 × 10^5^ cells l^−1^ on 15 July (Table 1 and black line in Fig. 3).

It could be argued that the bloom was simply related to advection of algae from open waters outside the fjord to the site below the ice, but this is contradicted by the simultaneous decrease in salinity (Fig. 1e). Under-ice salinities decreased due to rapid ice melt and freshwater drainage from melt ponds, and this signal would have been strongly dampened with any inflow of higher saline water. Furthermore, under-ice surface (1 m) water temperatures decreased from 0.3 °C on 9 July to 0.07 °C on 12 July (data not shown) whereas surface waters from areas with no ice cover are warmer and typically 1–2 °C at this time of year^9,29,30^. The under-ice algae were clearly acclimated to low light conditions given the relatively low under-ice *E*_k_ values (mean of 75 µmol photons m^−2^ s^−1^) derived from ^14^C incubations (Fig. S1), in comparison to values for under-ice and open-water phytoplankton in other actively blooming populations in Arctic studies^11^. In addition, the presence of the haptophytes and the taxonomic composition of the under-ice phytoplankton community was different from the most abundant open water planktonic species in Young Sound fjord^31^.

The first record of a *Chrysochromulina* under-ice bloom was in the brackish Baltic Sea at a salinity of 5–6^19^. Similarly, the cells observed in this study were dominated by small (3–5 µm) rounded cells with two long flagella and in many cases a long and coiled haptonema typical of e.g., the toxic mixotrophic species *Prymnesium polylepis* and *Chrysochromulina leadbeateri*. The under-ice bloom provided up to 740 mg C m^−2^ day^−1^ of new production (Fig. 2b, blue bars) and was primarily dominated by mixotrophic haptophytes with a relative abundance of 64%, as compared to 36% relative abundance of typical strictly autotrophic phytoplankton species (Fig. 3). This productivity was four times higher than previously measured in open water phytoplankton blooms in Young Sound^8,11^. This documents the importance of such under-ice blooms driven by mixotrophic haptophytes (Fig. 3). We suggest that under-ice blooms driven by mixotrophic algae are an overlooked succession phenomenon in nutrient-depleted and freshwater-influenced environments in the Arctic, which could imply a shift in the dominant algae species driven by freshening.

The proposed link between high under-ice productivity and mixotrophy is further supported by the low bulk NO_x_ concentration in sea ice and under-ice water (< 0.4 µM; Fig. 2a,b, dashed black line). Assuming that nutrient uptake followed the standard Redfield-Brzezinski ratio of 106C:16N:15Si:1P, NO_x_ appears (N:P ratio < 1) to have been deficient both in sea ice and seawater (Fig. 2a,b, dashed black lines). Our observations therefore challenge the classical view that limiting nutrient concentrations control algal biomass, as we observed an acute 9-day long under-ice bloom initiated under these nutrient-limited conditions. This suggests that mixotrophy is a strategy that might provide a growth benefit in these Arctic brackish waters where nutrients are not stoichiometrically balanced. This is supported by the measurements of biomass-specific productivity and maximum quantum yield (*F*_*v*_*/F*_*m*_) in Table 1, which verify that microalgae collected from sea ice and under-ice water were viable and photosynthetically active under these nutrient-limited conditions. The ongoing freshening of Young Sound and the Arctic Ocean with increased stratification and reduced vertical nutrient fluxes can accordingly promote mixotrophic-dominated algae blooms. Fundamental knowledge about the mechanisms driving these sub-ice blooms is important if we want to forecast their role and future prevalence. Therefore, we suggest that further expeditions aim to improve the understanding of these extensive under-ice blooms driven by potentially toxic mixotrophic haptophyte species. The consequences for the Arctic marine ecosystems and carbon budgets of the shift in the dominant algae to a potentially mixotrophic haptophyte species are important focus areas for future studies.

## Materials and methods

### Abiotic parameters

Sampling was conducted during the sea ice melting season in Young Sound, NE Greenland (74° 16′ 50 N, 20° 18′ 43 W) in 2017. The under-ice PAR measurements were obtained from a new and calibrated Odyssey PAR censor (Dataflow Systems) mounted on a cable and placed 2.4 m below the ice between 1 June and 7 July 2017. Under-ice PAR time-series were extended to 15 July by applying the transmittance of the ice (0.09), determined as ratio between measured CTD and surface PAR from the nearby Zackenberg Research station (https://data.g-e-m.dk/). Sea ice cores were collected on four occasions (7, 9, 12 and 15 July 2017) using a MARK II coring system (Kovacs Enterprises). All samples were collected within a 10 m^2^ (3.2 m × 3.2 m) quadrat. Triplicate cores were collected for physical and chemical samples during each sampling and cores for biological parameters were sampled in duplicate. The under-ice seawater samples were collected on four occasions (8, 9, 12 and 15 July 2017) using a Niskin (General Oceanics) water sampler at three depths: 1 m, 15 m and 30 m. The sea ice cover broke up on 15 July after which the ice floes were transported out of the fjord by winds and tides. Open seawater samples were thereafter collected twice (16 and 17 July 2017) also at three depths: 1 m, 15 m and 30 m. Vertical profiles of temperature, salinity, irradiance (photosynthetically active radiation; PAR) and fluorescence in the under-ice and open seawater column were measured using a CTD profiler (Seabird SBE19plus) equipped with additional sensors for Photosynthetic Active Radiation (Biospherical QSP-2350L Scalar sensor).

The air temperature was measured 2 m above the melt pond, and vertical profiles of temperature within the ice were measured using a thermometer (Testo). Light attenuation (used to calculate in situ primary production of the sea ice) was determined using standard methods^32^. The sea ice sections were placed in plastic containers and transported back to the laboratory in thermally insulated boxes. To determine bulk concentrations of O_2_ and TCO_2_ (TCO_2_ was used in this study to calculate potential primary production; Fig. S1) in sea ice, three sea ice cores were cut into 10 cm sections and placed immediately in gas-tight plastic bags^33^ and transported back to the laboratory in thermally insulated boxes. TCO_2_ and O_2_ were determined using standard methods^33,34^. Average values of triplicate samples are reported.

In the laboratory, sea ice sections were weighed for later determination of density and then melted in the dark over a 2-day period at 3 ± 1 °C. Conductivity of melted sea ice was measured (Thermo Orion-star with an Orion 013610MD conductivity cell) and converted to bulk salinity^35^. Average values of triplicate samples are reported.

Unfiltered under-ice and open seawater was transferred by gastight Tygon tubing to tubes (12 ml Exetainer) for TCO_2_^33^ and O_2_^34^ analysis following the standard analysis procedure.

### Chemical and biotic parameters

Triplicate melted sea ice cores (10 cm sections) and under-ice and open seawater samples (1 m, 15 m, 30 m) were analysed for inorganic nutrients (PO_4_^3−^, NO_2_^−^ + NO_3_^−^ (NO_x_) and NH_4_^+^), Chlorophyll *a* (Chl *a*) and photosynthetic activity. Duplicate samples were collected for primary production and algal abundance. Melting of sea ice was carried out following the protocols of Lund-Hansen et al.^17^ to avoid osmotic shock during ice melt, and photosynthetic and photobiological parameters are therefore maximum values.

Potential primary production was determined in melted sea ice cores cut into three sections (top, middle and bottom), and under-ice and open seawater at three depths (1 m, 15 m and 30 m) incubated for 3.15–6.49 h at 1 ± 1 °C at eleven laboratory light intensities (299 ± 128, 131 ± 35, 97 ± 47, 62 ± 28, 47 ± 24, 35 ± 19, 27 ± 15, 17 ± 8, 13 ± 7, 8 ± 6, 0 µmol photons m^−2^ s^−1^) using the H^14^CO_3_^−^ incubation technique^36^, and following the primary production procedure^37^. The potential primary production (µg C l^−1^ h^−1^) measured in the laboratory for different sea ice depths was plotted against the eleven laboratory light intensities and fitted to the function^38^ (Fig. S1). An estimate of primary production was calculated for each hour and sea ice depth using hourly in situ PAR hourly averages received through the Greenland Ecosystem Monitoring Programme (https://data.g-e-m.dk/). Average values of duplicate samples are reported.

For determining Chl *a* concentrations in melted sea ice and under-ice and open seawater, a known volume was filtered onto 25-mm Whatman GF/F filters. The filters were extracted for 18 h in 96% ethanol^39^ and analysed fluorometrically (TD-700, Turner Designs) before and after addition of 200 µl of a 1 M HCl solution. The fluorometer was calibrated against a pure Chl *a* standard (Turner Designs). Average values of triplicate samples are reported.

For determining inorganic nutrient concentrations, triplicate samples of melted sea ice, under-ice water and seawater without ice were filtered through 0.45 µm filters (Q-Max GPF syringe filters) into 6.5 ml scintillation vials and frozen (− 19 °C) for further analysis of PO_4_^3−^, NO_x_ and NH_4_. The concentrations were measured on a Seal QuAAtro autoanalyzer using standard colorimetric methods. Average values of triplicate samples are reported.

Pulse amplitude modulated (PAM) fluorometry (Phyto-PAM Phytoplankton Analyzer, Heinz Waltz GmbH, Germany) was applied to measure algae photoacclimation and photosynthetic activity in triplicate samples from melted sea ice core sections (10 cm), under-ice, and at depths (1 m) in open seawater with no ice. All PAM measurements were conducted in the dark to keep algae dark-incubated and the photosynthetic parameters were derived by means of the rapid light curve technique^40^ and the curve-fitting algorithm^41^. Average values of triplicate samples of maximum quantum yield (*F*_*v*_*/F*_*m*_) are reported. Temperature in a snow bath with measuring unit was controlled by an US-T Temperature Control Unit and kept close to 1 °C. Temperature of the samples was measured by a thermometer (LT-101, Lab Thermometer IP65, TFA Dostmann/Wertheim).

For algal abundance measurements, samples of melted sea ice and under-ice and open seawater were fixed with acidic Lugol’s solution (final concentration of 1%) and stored dark and cold (3 ± 1 °C). Algal cells were identified and counted from light-microscopy analyses using the Utermöhl sedimentation technique. Algae cells were analysed within 2 months of collection, average values of duplicate samples are reported.

Using light microscopy, it is not possible to identify small naked flagellates such as the haptophytes to species level. The taxonomy of the haptophytes is currently under revision, and the bloom forming mixotrophic species formerly known as *Chrysochromulina polylepis,* which is a typical haptophyte with a long and coiling haptonema, has been transferred to the genus *Prymnesium*.

## Supplementary Information

Supplementary Figure S1.

## Data Availability

The data produced during the current study are available from corresponding author based on a reasonable request.
