# Supplementary figures and images for: An under-ice bloom of mixotrophic haptophytes in low nutrient and freshwater-influenced Arctic waters
Source: Sci Rep. 2021 Feb 3;11:2915. doi: 10.1038/s41598-021-82413-y (PMC7858608; doi:10.1038/s41598-021-82413-y)

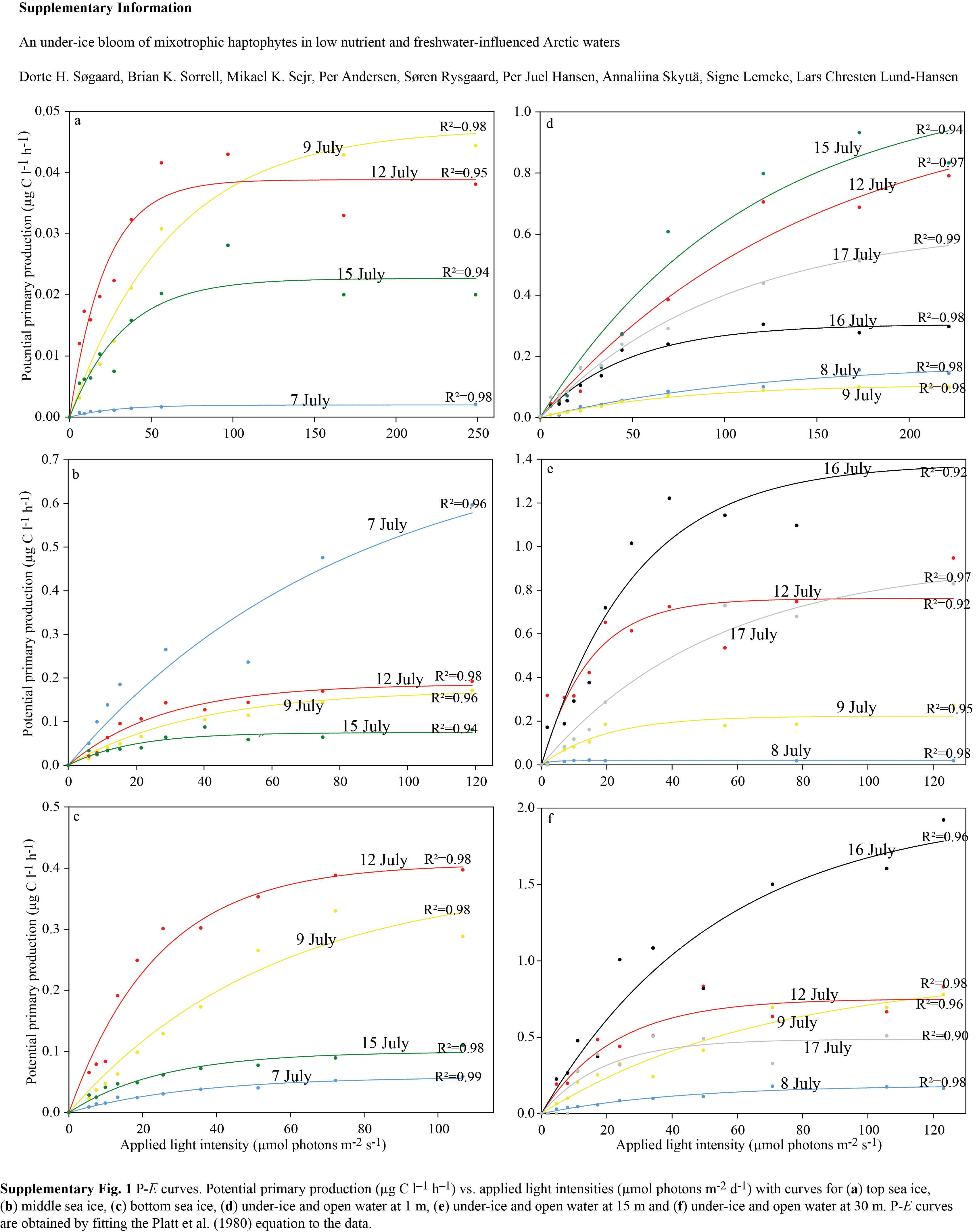

Supplement: Supplementary file 1 — Supplementary Figure S1. [file 41598_2021_82413_MOESM1_ESM.png]
